# Resting-state connectivity stratifies premanifest Huntington’s disease by longitudinal cognitive decline rate

**DOI:** 10.1038/s41598-020-58074-8

**Published:** 2020-01-27

**Authors:** Pablo Polosecki, Eduardo Castro, Irina Rish, Dorian Pustina, John H. Warner, Andrew Wood, Cristina Sampaio, Guillermo A. Cecchi

**Affiliations:** 1 IBM T.J. Watson Research Center, Yorktown Heights, Yorktown, NY USA; 2 CHDI Management/CHDI Foundation, Princeton, NJ USA

**Keywords:** Machine learning, Huntington's disease

## Abstract

Patient stratification is critical for the sensitivity of clinical trials at early stages of neurodegenerative disorders. In Huntington’s disease (HD), genetic tests make cognitive, motor and brain imaging measurements possible before symptom manifestation (pre-HD). We evaluated pre-HD stratification models based on single visit resting-state functional MRI (rs-fMRI) data that assess observed longitudinal motor and cognitive change rates from the multisite Track-On HD cohort (74 pre-HD, 79 control participants). We computed longitudinal performance change on 10 tasks (including visits from the preceding TRACK-HD study when available), as well as functional connectivity density (FCD) maps in single rs-fMRI visits, which showed high test-retest reliability. We assigned pre-HD subjects to subgroups of fast, intermediate, and slow change along single tasks or combinations of them, correcting for expectations based on aging; and trained FCD-based classifiers to distinguish fast- from slow-progressing individuals. For robustness, models were validated across imaging sites. Stratification models distinguished fast- from slow-changing participants and provided continuous assessments of decline applicable to the whole pre-HD population, relying on previously-neglected white matter functional signals. These results suggest novel correlates of early deterioration and a robust stratification strategy where a single MRI measurement provides an estimate of multiple ongoing longitudinal changes.

## Introduction

The detection of treatment effects in clinical trials is partly limited by the individual differences in the population under study. In neurodegenerative disorders, enrichment strategies to decrease sample heterogeneity and identify subjects likely to respond to treatment are important for the efficiency and success of clinical trials, especially for early interventions^1^. Given the high dimensionality of neurological data, computational approaches including machine learning techniques appear particularly promising for detecting individual differences in clinical populations by integrating distributed information, providing stratification of subjects for predictive or enrolment purposes^2–4^.

Huntington’s disease (HD) is a neurological disorder caused by an expansion in the cytosine-adenine-guanine (CAG) trinucleotide repeat region in the huntingtin (HTT) gene. The earliest symptoms are related to changes in mood and cognitive ability, followed by lack of motor coordination, unsteady gait, and chorea, ultimately leading to dementia and death^5^. If we take into consideration that clinical damage associated with the disease is potentially irreversible, early intervention is imperative. For this to happen, trials with patients at early stages should be tailored towards individuals who will develop a steep deterioration, since testing experimental early treatments on stable individuals can produce misleading results^6^.

The availability of genetic tests has enabled large neuroimaging studies, such as PREDICT-HD^7^ and TRACK-HD^8,9^, which have followed patients and controls over several years, even before motor-based diagnosis. These cohorts provide opportunities for moving beyond descriptive statistical observations toward assessments that provide predictions at an individual level^4^. (Note that in the context of predictive modelling, “prediction” refers to the ability to make assessments on previously-unencountered individuals, not necessarily forecasts of future events). Cross-validated strategies are a step toward that goal, where different sets of subjects are used for training a model and for testing its individual predictions^10^. In that sense, it is critical that the signals distinguishing individuals are reliable across successive measurements that are repeated on time scales that are short with respect to the expected rate of change^11^, but also that learned patterns are shown to be robust, e.g. valid across sub-cohorts from different imaging sites^12^.

In neuroimaging, structural correlates of HD such as striatal atrophy have received most attention^13^. Resting state functional MRI (rs-fMRI) is a more recent development^14^ and, since then, most analyses have been restricted to specific subnetworks or considered few regions, or seeds. However, functional brain networks are scale-free^15^, i.e. their degree distributions follow power laws spanning several orders of magnitude. This phenomenology can only be observed on networks with a large number of nodes such as voxel-level resolution networks. In addition, functional signals and networks of neurobiological significance have been recognized recently in white matter^16–19^. Therefore, previously-overlooked correlates of disease progression may be found by analyses designed to reflect these major aspects of functional networks.

Track-On HD^20^ is an extension of the TRACK-HD study that included yearly rs-fMRI data for up to 3 years in more than 200 pre-manifest HD (pre-HD, i.e. before diagnosis based on motor symptoms) subjects and healthy controls. Images are accompanied by a wealth of cognitive and motor measures, most also acquired during preceding TRACK-HD visits, in combination spanning up to 7 yearly measurements. Such a rich dataset allows for the estimation of subtle longitudinal changes in cognition and motor control, allowing subjects to be compared to their own performance baselines, and enabling analyses of population heterogeneity for a stage at which systematic brain differences from controls are mild^21^. In addition, because performance on different tasks might rely on the same affected neural systems, it is possible to identify combinations of measures for which longitudinal changes covary, potentially boosting behavioural signal-to-noise and isolating common brain structures responsible for cognitive decline.

**Figure 1:**
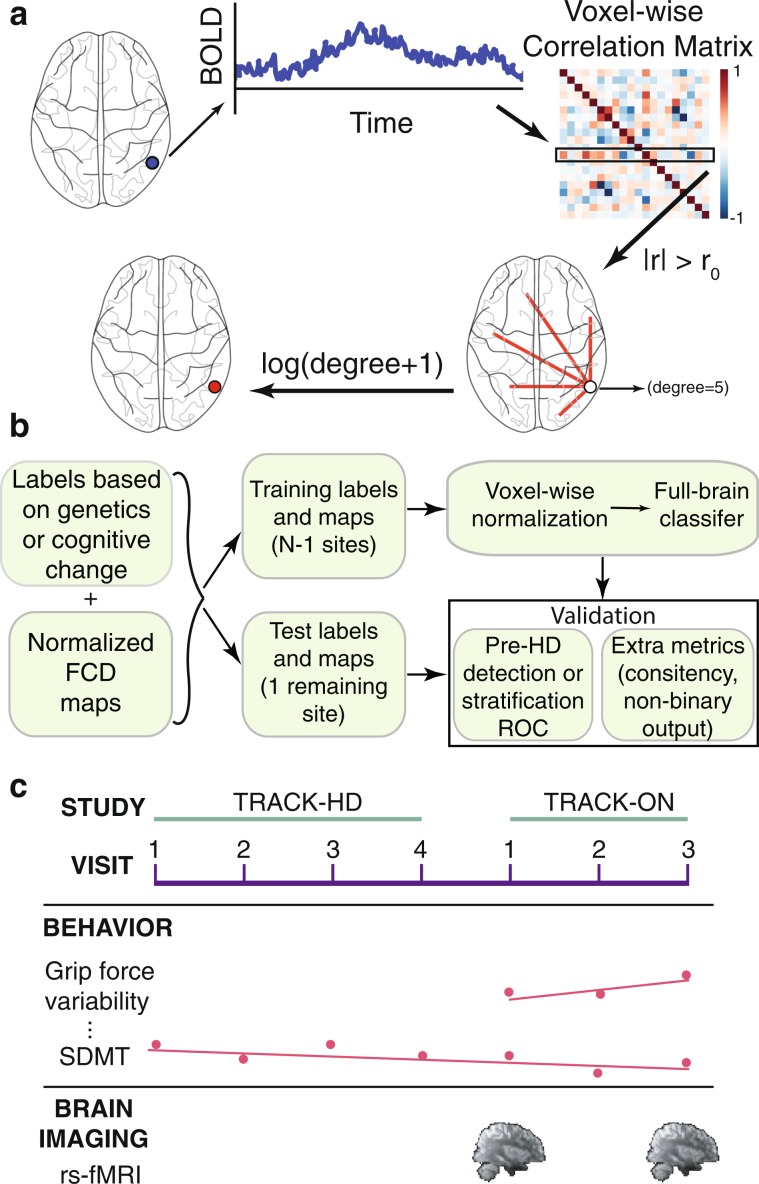
Brain measures, models, and cohort. (**a**) Functional connectivity density (FCD) maps. A voxel-wise correlation matrix is computed from BOLD time courses and functional links are defined as above-threshold correlations. A voxel’s degree, defined as the number of its associated links, is a simple measure of centrality. We refer to the logarithm of degree (plus one) as FCD. (**b**) Multivariate cross-validated models. Subjects were assigned a label defined by a measure of cognitive/motor decline after correcting for healthy age/sex expectations (fast vs slow decline, excluding intermediate subjects during training), or by genetics (pre-HD vs control). Subjects from three of the four imaging sites (all but one) were included in the model training set. Performance of the model was estimated on subjects from the remaining site, and the process was iterated for every site (leave-one-site-out cross-validation, LOSO-CV). Test-site validation was quantified by the area under the receiver operating characteristic (ROC) curve (AUC). Additionally, for cognitive stratification, the Spearman rank correlation of the continuous output of classifiers vs cognitive decline rates was estimated using all subjects from test sites. (**c**) Patient cohort. Participants belonged to the Track-On HD study. Two rs-fMRI samples were used per subject. Longitudinal change in performance on 10 cognitive and motor tests was evaluated, including measurements from the immediately preceding TRACK-HD study.

Here, we evaluate the potential of single rs-fMRI scans for stratification of pre-HD individuals with respect to cognitive/motor decline, which has never been tested. We built functional connectivity density (FCD) maps (Fig. 1a) from a Track-On HD cohort of 153 subjects (74 pre-HD, 79 healthy controls) at the voxel-level. We first established test-retest reliability of multivariate FCD measures. We then trained cross-validated predictive models (Fig. 1b) to segregate pre-HD subpopulations according to cognitive/motor longitudinal trajectories (Fig. 1c) along multiple dimensions. To establish robustness of predictive power, we used a leave-one-site-out cross-validation (LOSO-CV) approach. The resulting models suggest a stratification strategy that considers disease progression in terms of longitudinal rates of change along different cognitive dimensions, and where a single MRI scan provides such information, otherwise measurable over years.

## Methods

### Participants

We included 153 participants from the four Track-On HD study sites in this analysis, belonging to one of two groups: 74 individuals without manifest HD (before diagnosis by a physician based on motor symptoms) but carrying the mutant HTT gene and 79 roughly age- and sex-matched controls (see Table 1 for demographics).

**Table 1:** Cohort demographics.

	Controls	Pre-HD
N	79	74
Sex (Female)	30	34
Age (mean ± STD)	49 ± 11	42 ± 10
CAG	—	43 ± 2
CAP	—	48 ± 8
Expected years to disease onset	—	11 ± 4 (minimum: 5)

CAG: number of CAG repeats in the HTT gene. CAP: CAG-age-product^66^, a measure of disease burden given by the product of a subject’s number of excess CAG repeats and their age. Here we use the formula CAP = 100 × AGE × [(CAG − 35.5) ÷ 627], i.e. normalized so that score at disease onset (diagnosis based on motor symptoms) is approximately 100.

Exclusion criteria for analysis were as follows: manifest disease at any point before MRI acquisitions, age below 18 or above 65 (unless previously part of the TRACK-HD study), history of major psychiatric, neurological, or medical disorders, or severe head injury. These criteria are part of the Track-On HD study^20^. In addition, we excluded left-handed individuals, as known rs-fMRI lateralization differences in motor regions^22^ are outside the focus of this analysis, as well as those with fewer than two visits with rs-fMRI scans. Furthermore, we excluded subjects based on scan motion or unsatisfactory registration to standard template (see below, and Supplementary Table S1 for detailed numbers).

The Track-On HD study was approved by local ethics committees (Leiden site: Medical Ethical Commission of Leids Universtais Medisch Centrum; Vancouver site: Clinical Research Ethics Board at the University of British Columbia; London site: National Research Ethics Service of the National Health Service (NHS) London; Paris site: Comité de Protection des Personnes Ile De France VI, Pitié-Salpêtrière Hospital) and all participants gave written informed consent according to the Declaration of Helsinki. All methods were performed in accordance with the relevant guidelines and regulations.

### Cognitive/motor measures

On each visit, participants (both controls and pre-HD patients) were administered several motor and cognitive tests. For consistency with previous studies^20^, the following tests were considered: Symbol Digit Modalities Test (SDMT), self-paced tapping, grip-force variability, map search, Stroop test, spot change test, cancelation test, mental rotation, indirect circle tracing, and counting backwards. Detailed description of tasks and their outcome measurements is provided elsewhere^20^ (see Supplementary Information for a brief summary). Tests were administered on each yearly visit in most cases (up to 3 in Track-On HD). Several tests were also included in the immediately preceding visits from the previous TRACK-HD study^8^, in which case up to seven time points were available (Fig. 1c, distribution of visits for each task in Supplementary Fig. S1).

To quantify longitudinal cognitive change for asymptomatic HD patients, we computed the slope of longitudinal performance change for each task. The underlying change is not necessarily linear; however, slopes are used as the simplest measure of subtle decline given the few time points per subject and measurement noise. All sessions in which a given subject was administered a test were included in the slope estimation, including those preceding fMRI acquisitions.

For consistency, we defined the sign of all task measures such that a negative longitudinal slope would indicate decline.

As we were interested in cognitive decline beyond the effects of healthy aging sex or age, we removed variance associated with these covariates. Linear effects of sex and age were estimated and removed using data from healthy controls.

### Robust principal components for cognitive/motor measurements

Different tasks involve overlapping skills and brain circuits. Therefore, their longitudinal slopes share with each other a portion of inter-subject variability (i.e., they covary). To find measures of degeneration along different *effective* dimensions (that is, reflecting decoupled aspects deterioration), we computed decorrelated combinations of slopes via Principal Component Analysis (PCA), based on their covariance matrix. To reduce sensitivity to the presence of outliers, we used Minimum Covariance Determinant^23^, a robust method to estimate the covariance matrix. Units of the slopes of different tasks are different, so we also normalized slopes of each task by its interquartile range to produce a dimensionless quantity that could be combined with the other tasks.

We performed the PCA procedure twice: 1) First, both pre-HD subjects and controls were included, and the slopes were corrected (see below) for effects of normal aging and sex estimated from healthy subjects (using the age at the time of acquisition of the first functional image). This allowed a visualization of pre-HD and control trajectories, to confirm longitudinal differences were observable. 2) A second PCA included only slopes from pre-HD subjects, to best capture heterogeneity within this population. These are the principal components (PCs) we used for stratifying the pre-HD population (see “Discretization of cognitive/motor trajectories” below, and Supplementary Fig. S2 for a flowchart).

### MRI data Acquisition

3T MRI data from 2 different scanner systems (Siemens Tim Trio and Philips Achieva) distributed across four acquisition sites were acquired, as described elsewhere^8,20,24^, including multiple design measures before and during acquisition to maximize data homogeneity across sites^20^.

In brief, T1-weighted image volumes were acquired using a 3D MPRAGE acquisition sequence with the following imaging parameters: TR = 2200 ms (Siemens)/7.7 ms (Philips), TE = 2.2 ms (S)/3.5 ms(P), FA = 10° (S)/8° (P), FOV = 28 cm (S)/24 cm (P), matrix size 256 × 256(S)/224 × 224(P), 208(S)/164(P) sagittal slices to cover the entire brain with a slice thickness of 1 mm (no gap 1 mm isotropic voxels).

For rs-fMRI, whole brain volumes were acquired with a TR of 3 s using a T2*-weighted echo planar imaging (EPI) sequence with the following parameters: TE 30 ms, FOV 212 mm, flip angle 80°, 48 slices in ascending order (thickness: 2.8 mm, gap: 0.5 mm, in plane resolution: 3.3 × 3.3 mm) and bandwidth of 1906 Hz per Px. 165 time points were acquired for 8:20 minutes. All data passed visual inspections centralized by IXICO.

### MRI data Processing

fMRI pre-processing used FSL^25^ parallelized for large-scale computation using Nipype^26^. The first 5 time points of the fMRI time series were discarded to exclude unsteady acquisitions. Slice-timing correction was applied followed by rigid realignment to the volume in the middle of the series (motion-correction). Subjects with more than 10 frames with relative displacement (as computed by FSL’s *mcflirt* command) above 0.6 mm were excluded (4 subjects, Supplementary Table S1). Physiological (respiration, heart rate, etc.) as well as motion-associated noise was corrected using the tCompCor method^27^ that identifies a noise-dominated mask and removes its associated temporal variance. We deliberately avoided imposing an anatomical white matter (WM) mask to preserve recently recognized WM signals of neural origin^16,18,19,28,29^. On inspection, noise masks isolated cerebrospinal fluid and head edges. Finally, the time series of each voxel was band-pass filtered (0.01–0.16 Hz) to remove drifts and high-frequency noise (see Supplementary Fig. S3 for a flowchart).

In-session anatomical volumes underwent brain extraction (BET) followed by non-linear normalization to MNI space (FNIRT). All EPI time series were then rigidly co-registered to the in-session anatomical volume and non-linearly normalized to MNI standard space using the same transformation, preserving original voxel size (3.3 mm isotropic).

Acceptability of the concatenated registrations was verified as in other large sample studies^30^. Session-specific average volumes for each participant and a group average template were computed. Participants whose time-averaged EPI volume had a correlation with the template that deviated more than 3 standard deviations from the population were excluded from the analyses upon visual re-inspection.

### Feature extraction

We computed voxel-level functional networks (Fig. 1a) as follows: (1) pair-wise Pearson correlation coefficients were calculated between voxels; (2) correlations above 0.7 constituted links, as in previous studies^31^.

For each voxel, we computed the log-degree, defined as *log(degree + 1)*, where degree is defined as the total number of links. Each resulting log-degree map was normalized by the median value across all voxels as baseline reference. We refer to the resulting maps as FCD maps (see Supplementary Fig. S4 for a flowchart).

Univariate comparisons (described below) rely on local signals, so FCD maps were locally enhanced by Gaussian smoothing (FWHM = 2.5 voxels). For multivariate classifications, which pool signals together, no smoothing was used.

The above procedure was repeated to compute separate FCD maps from each individual at each visit.

### Multivariate classifications

Using FCD maps, multivariate classification between 2 groups (pre-HD vs controls, or fast vs slow-decline) was performed using logistic regression with elastic net regularization, using a LOSO-CV scheme (Fig. 1b, see flowchart in Supplementary Fig. S5), to ensure robustness of predictions across imaging sites. Elastic net regularization is a penalty term that automatically performs variable selection, reducing overfitting. Unlike other variable selection terms such as LASSO, elastic net can select more features than the number of training samples (critical when the number of features is much larger than available samples), and is more stable when correlated features are present. Logistic regression was chosen over other linear classifiers due to good optimization convergence when combined with elastic net penalty using a SAGA optimizer^32^ on similar datasets^33^, but model performance is similar to a standard support vector machine. Optimal value of regularization parameters was determined within each training set via internal 5-fold cross-validation (i.e., nested cross-validation).

For classifications of pre-HD vs controls, FCD maps were linearly corrected for healthy aging and sex during model training, using weights learned from controls in the training set.

The metric of evaluation of binary classifiers was the area under the receiving operator characteristic (ROC) curve (AUC). This metric has the advantage that it is a measure of a continuous output, independent of the choice of a decision threshold, and robust to imbalanced classes. We computed the AUC for each test site and averaged them. For robustness of the estimation to outliers, we repeated the computation with bootstrap resampling (random resampling with replacement, 10^5^ resamples) of the test subjects, and reported the median value. In the case of stratification models, it should be noted that the AUC tests whether a pattern of ongoing decline is successfully learned, but not its application to the general pre-HD population. For that we performed a second validation (see “Validation of continuous classifier output for pre-HD stratification” below).

All predictive modelling was implemented using scikit-learn^34^ and Lightning (http://contrib.scikit-learn.org/lightning/index.html) libraries in Python. Plots of results on the brain (and brain schematics used in figures) were generated using Nilearn^35^.

### Univariate correlations of FCD features with cognitive/motor decline

For tasks slopes and their PCs, we computed voxelwise Spearman correlations with FCD, which were converted to a t-statistic from which statistical significance was calculated, and multiple comparison correction was applied (false discovery rate, FDR, see below).

### Discretization of cognitive/motor trajectories

Exact values of task performance and their slopes are invariably noisy (see “Estimation of noise in cognitive slopes” in Supplementary Information for a quantification), but they can be used to isolate coarse subgroups of subjects, which has proven useful for longitudinal change in other disorders^36^. We defined three subgroups for each single task or PC projection: fast, intermediate, and slow, which corresponded respectively to subjects below, in-between, or above half a robust estimate of the standard deviation of decline away from the median. The robust estimate of the standard deviation was defined as the standard deviation of a normal distribution with the same interquartile range as the observed distribution of decline, making it insensitive to outliers. This threshold was chosen because, while being the same for all tasks, it divided the pre-HD population approximately into terciles. We performed cross-validated classifications between the extreme subgroups (fast and slow) on the basis of FCD maps (stratification models). Subjects with a slope or PC projection in-between these extreme ranges were not included in the classification of the two subgroups. This was the case for roughly one third of the subjects, the exact number depending on the PC or task (Supplementary Table S5). The subgroups used for training/testing the models did not differ in the number of visits used for computing the slopes (Supplementary Table S4).

### Validation of continuous classifier output for pre-HD stratification

We propose that brain signatures based on extreme subgroups could be used to provide information about ongoing decline in any subject of the population, including those in the intermediate decline group. As a validation of this proposed application, we computed the Spearman rank correlation between the continuous output of the stratification models (signed distance to the decision hyperplane) and cognitive decline rates using all subjects from each test site. Spearman correlations computed within each of the four test sites were Fisher z-transformed, averaged over sites, and then inverse-transformed back to r-space, as is common practice when pooling together correlation coefficients^37^.

### Integration with expectations from genetics

To quantify the variance in cognitive decline rates explained by CAG repeats, we computed the Spearman correlation between them. We also studied whether combining CAG repeats as a feature with FCD maps could improve predictive power in stratification models.

### FCD test-retest reliability

We studied the consistency of FCD maps within individuals in two different ways.

First, we studied the correlation between baseline and follow-up FCD maps, in terms of their power to identify individuals (“fingerprinting”), similar to others^11^. For the baseline FCD map of each individual, we computed its Pearson correlation (across voxels) to the follow-up FCD map of each individual, and ranked these correlations (153 correlations). We stored the rank of the correlation between the same subject’s baseline and follow-up scan, a measure of how similar the two were relative to similarity to other subjects. The resulting distribution of 153 stored same-subject ranks was compared to chance levels.

Second, using LOSO-CV, we tested the within-subject consistency of predictions of a multivariate classifier trained to distinguish a neurologically relevant signature: that of pre-HD subjects vs. controls. We computed a transition matrix with the probability of a follow-up classification outcome given a baseline classification outcome, and compared the observation to chance levels (see “Statistical Significance Analyses” below).

### Removal of local atrophy effects by voxel-based morphometry (VBM)

As a control for the effects of local atrophy, we used an estimation of grey matter concentration from voxel-based morphometry, as proposed by Oakes and colleagues^38^. The ‘optimized’ VBM protocol^39^ of the FMRIB Software Library (FSL)^25^ was used to evaluate grey matter patterns, as detailed in a previous study on the TRACK-HD cohort^40^. For each subject a T1-weighted image was used along with masks estimated from concurrent T2-weighted volumes to do brain extraction of the raw T1-weighted volume. The resulting images were then segmented into 3 tissue partial volumes (grey matter, white matter, and cerebrospinal fluid concentrations) that represent the probability of each voxel belonging to a given tissue. Grey matter concentration (GMC) volumes of each subject/visit were nonlinearly normalized to the MNI standard space using FSL’s FNIRT routine (without normalization by the Jacobian determinants). The resulting GMC images were down-sampled to the resolution of FCD maps using linear interpolation to preserve their range. For each voxel the linear effect of GMC on FCD across subjects was estimated (i.e., a voxelwise covariate). For each subject at each voxel, the expected FCD based on the local GMC was subtracted from the observed FCD. The residuals constituted local-atrophy-controlled FCD maps. We used this atrophy-controlled FCD maps in multivariate detection of pre-HD (vs controls), and stratification models, for comparison of the resulting predictive power, and their associated weight maps. In addition, we evaluated full-brain GMC maps in terms of their multivariate predictive power to differentiate pre-HD vs control subjects, and stratification of the population with a LOSO-CV scheme, identically as done with FCD maps.

### Removal of site variability

To minimize potential effects of imaging site on learned FCD patterns, we removed the associated variability from FCD maps using ComBat^41^, a technique originally proposed to remove batch effects in gene expression data and recently shown to outperform several others for harmonization of maps from different MRI modalities^42,43^. For each voxel, ComBat estimates a site-specific mean value and site-specific scaling factor. Importantly, while ComBat allows for the specification of biological variates of interest (subject group or subgroup, age, sex, etc.) to best preserve such variance, we chose not to include them in the model in order to guarantee information about classification labels would not leak into the corrected FCD maps. We used a Python implementation of the original method (https://github.com/brentp/combat.py).

### Removal of motion associations

Rigid motion correction and motion artefact removal with tCompCorr had been applied to the fMRI time series. However, no method is able to completely mitigate motion-related artefacts^44^, so we additionally controlled in stratification models for the effects of motion by removal of motion variability across subjects in each feature, as proposed by others^45^. The mean framewise displacement was used as a quantifier of head motion^44^, as provided by FSL’s *mcflirt*, which calculates the average voxel displacement between successive time points in terms of rigid transformations over a spherical, brain-sized region of interest. To remove motion artefacts while preserving the neurological signals potentially correlated with motion in pre-HD subjects, the linear weight of motion on each feature was learned from healthy controls, who had a motion distribution very similar to the pre-HD population (see Results, “Robustness of FCD patterns to non-functional covariates”). We detrended FCD features with respect to head motion in the pre-HD population using these weights, and compared the resulting stratification model and FCD patterns to those without this additional motion control.

### Statistical significance analyses

All voxel-wise univariate statistical tests for between-group comparisons were analysed with non-parametric Mann–Whitney U tests. We controlled for multiple comparisons by estimating a false discovery rate (FDR) through the Benjamini–Hochberg procedure^46^.

Null distributions for assessing significance of cross-validated classification results were computed via permutation of test subject labels (10^5^ randomizations, implemented in Python) within each site. Similarly, null distributions for the Spearman correlations between decline rates and distance to the decision hyperplane were obtained via permutation of test subject labels (10^5^ randomizations) within each site.

For test-retest reliability analyses (transition matrix), we computed a null distribution of within-subject consistency of classifier predictions: classifier predictions of each subject’s baseline FCD were compared with a distribution of follow-up visit predictions of randomized subjects that belonged to the same group (group-preserving permutations of the second sample, 10^5^ permutations). This ensured the null distribution samples contained the same proportion of each possible prediction for each group and visit as in the observed case, while only the within-subject correspondence between baseline and follow-up classification varied.

## Results

Using data from the Track-On HD cohort (Table 1), we first evaluated the test-retest reliability of full-brain FCD maps (46667 voxels) and then evaluated models that stratify the pre-HD population by longitudinal cognitive decline based on single-visit FCD maps.

### Test-retest reliability of FCD maps

**Figure 2:**
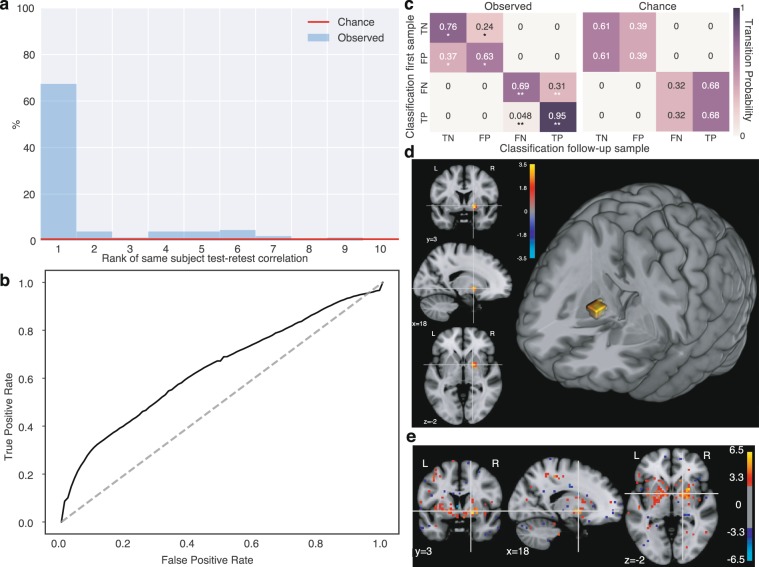
Test-retest reliability of FCD measures in pre-HD. **(a)** Correlation of test and retest FCD within subjects. For each subject’s baseline scan, we computed the correlation (across brain voxels) of the FCD map with that of the follow-up FCD map of all 153 subjects and ranked them. The histogram shows that, for 67% of subjects, the top-ranked correlation with follow-up FCDs was with their own follow-up, identifying them. Red line: 0.65% (i.e., 1/153) chance level. X axis is truncated at 10 for visibility. **(b)** Multivariate detection of group differences. ROC curve of a full-brain classifier of pre-HD vs controls (LOSO-CV). AUC: 0.64 (p = 0.00082, permutation test). Group differences are detectable but mild, compatible with heterogeneity. **(c)** Test-retest reliability of group differences. Correct and incorrect classifications are highly reliable, suggesting they are determined by consistent heterogeneity. Left: Matrix indicating probability of classification outcome of a follow-up FCD sample, given that of the baseline sample. Right: Chance level matrix (permutation test, see Methods). Both hits and errors are reliable within subjects. *: p < 10^−3^, **: p < 10^−5^ (group-preserving two-sided permutation test, see Methods) TN: true negative, FP: false positive, FN: false negative, TP: true positive. **(d)** Univariate FCD group differences. Single cluster on the globus pallidus and interface with the putamen (two-sided Mann–Whitney U test, FDR q < 0.05). Red: pre-HD > controls, Blue: pre-HD < controls. Units: logarithm of corrected p-value. **(e)** Mean maps of classifier weights reveal a bilateral globus pallidus cluster. Weights shown at an arbitrary threshold of 3, in standard units across voxels.

We validated FCD maps in terms of within-subject test-retest reliability. First, we evaluated the within-subject similarity of baseline and follow-up FCD maps, irrespective of a neurological signature of interest. For, this we evaluated the ability to identify (“fingerprint”) individuals on follow-up visits based on the correlations of follow-up and baseline FCD maps (Methods, “FCD test-retest reliability”). More that 67% of the baseline maps had their top correlation with the follow-up map from the same subject (Fig. 2a), two orders of magnitude above chance expectations (1 in 153, i.e. 0.65%, red line in Fig. 2a). Of the remaining cases, a large fraction had their same-subject correlation on the top rankings. This result suggests FCD maps reliably capture full-brain individual differences.

We then evaluated the reliability of FCD maps for reflecting a neurologically meaningful difference, that of pre-HD vs controls. Classification based on presence or absence of mutated HTT gene is ideal to study within-subject reliability: genetic labels have virtually no associated noise, and are strictly stable across visits. Moreover, because functional differences are mild in gene-carriers far from symptom onset^21^, the sensitivity to test-retest fluctuations is expected to be higher than for strong neurological signatures. We trained binary classifiers using sex- and age-corrected FCD maps validated using LOSO-CV to ensure robustness to site differences. The resulting AUC (0.64, p = 0.00082, permutation test, Fig. 2b) was compatible with mild but detectable signatures of pre-HD. Subjects classified correctly with the first FCD sample were highly likely to be classified correctly also with the second sample (95% consistency for pre-HD, p < 10^−5^; 76% consistency for controls, p = 0.0007 permutation test; Fig. 2c), indicating high consistency for correct classifications. Moreover, the whole transition matrix was dominated by the diagonal, so consistency of errors was also well above chance levels (63% for controls, p = 0.0007; 69% for pre-HD, p < 10^−5^, compare to chance matrix in Fig. 2c), suggesting misclassifications were driven by reliable FCD heterogeneity and not random noise. Together, the observed consistency suggests high test-retest reliability of multivariate FCD patterns from visits separated up to 2 years, and motivates the study of individual variation within the disease, which is consistently observed but not captured by group differences.

To visualize the FCD signatures of early degeneration in pre-HD, we first computed a univariate voxel-wise group comparison. Significant differences (Mann-Whitney U test, FDR corrected, q < 0.05) were localized in the globus pallidus and its interface with the putamen (Fig. 2d). Similar but more extensive results were obtained by inspection of the multivariate classification weights. A map of mean classifier weights (Fig. 2e) revealed the clusters were bilaterally symmetric. This suggests functional hyper-connectivity of the globus pallidus is a novel signature of pre-HD.

### Analysis of cognitive/motor longitudinal decline

To quantify cognitive/motor decline, we computed longitudinal slopes for 10 tasks (see Methods, Supplementary Information for task description). Steeper decline slopes of pre-HD subjects vs controls were observed for the Symbol Digit Modalities Test (SDMT, p = 0.009, Mann-Whitney U test), the Stroop test (p = 0.017, Mann-Whitney U test), indirect circle trace (p = 0.032, Mann-Whitney U test), and paced tapping (p = 0.014, Mann-Whitney U test) tasks (see Supplementary Table S2 for all tasks). Associations with healthy aging in controls were not significant (Supplementary Table S3). In pre-HD, longitudinal slopes were not significantly correlated with CAG repeats (Supplementary Table S3), but there were associations with CAP score for the counting backwards task (Spearman’s rho = −0.30, p = 0.01, t-test) and the Stroop test (Spearman’s rho = −0.25, p = 0.03, see Supplementary Table S3 for all tasks). It should be noted that age and CAG repeats were strongly anticorrelated in this population (Spearman’s rho = −0.63, p = 2*10^−9^, t-test). These observations indicate detectable longitudinal cognitive decline in pre-HD, but show weak associations with genetic burden.

**Figure 3:**
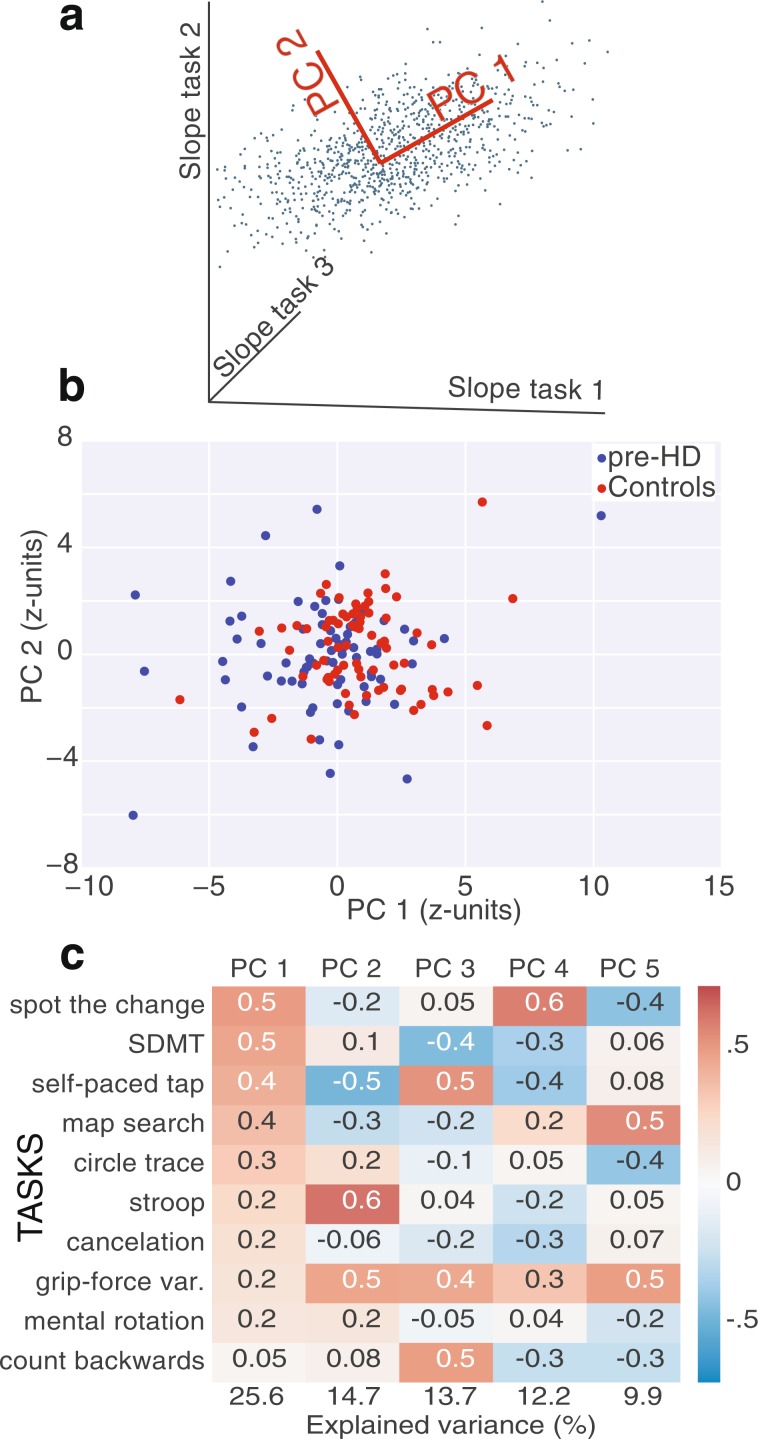
Longitudinal cognitive/motor decline in pre-HD. (**a**) Schematics of how PCA isolates effective directions of decorrelated change (PCs). (**b**) Projection of control and pre-HD slopes onto top 2 PCs of slopes (age/sex corrected) of performance from 10 tasks, showing differences along PC 1 (p = 2.2*10^−6^, two-sided Mann–Whitney U test). (**c**) Coefficients of the top 5 PCs, computed with slopes (healthy age/sex corrected) from pre-HD subjects only.

To isolate decorrelated components of decline, we performed PCA on the task slopes (Fig. 3a, see Methods). This first PCA was performed on age-corrected slopes of both pre-HD and controls (Fig. 3b). Strong longitudinal group differences became apparent along PC1 (p = 2.2*10^−6^, Mann–Whitney U test), consisting of a weighted sum of the most reliable cognitive tasks. Some pre-HD trajectories overlapped with controls (in the sense that those subjects were “stable”), but the asymmetry became prominent in ranges away from the mean, suggesting task slopes and their PCs are sensitive to pre-HD decline.

To study heterogeneity of decline within pre-HD alone, computed a second PCA using only subjects from this group (Fig. 3c). The top 5 PCs each captured more than or around 10% of the variance (i.e., the effective equivalent of 1 task out of 10), and the bottom 5 were disregarded.

### Stratification of pre-HD by longitudinal cognitive/motor decline rates using single-visit FCD

Single-task slopes (corrected for age and sex) and PCs were used for stratification models of the pre-HD population. For each such measure of decline rate, subgroups of fast, intermediate and slow-decline (or stable) subjects were defined (see Methods, flowchart in Supplementary Fig. S2, and Supplementary Table S5 for subgroup subject numbers). Slow and fast subgroups were used to train binary cross-validated classifier models using single-visit FCD maps as predictors (see Methods, flowchart in Supplementary Fig. S5). To establish robustness to site differences, we used a LOSO-CV scheme. First, we validated models in terms of their ability to effectively distinguish subjects from these extreme subgroups in test sites, excluding the intermediate subgroup. Then we studied the usefulness of the distance to the decision hyperplane as a proxy for the continuous value of cognitive decline. This key validation used test site subjects from all three subgroups. Below we summarize the results of this approach (complete results in Tables 2 and 3).

**Table 2:** Model accuracies.

Task	AUC	p-value	Spearman’s rho	p-value
SDMT	0.73	0.0023	0.41	0.00005
Cancelation	0.73	0.002	0.27	0.005
Paced tap	0.70	0.008	0.05	0.2977
Grip variability	0.61	0.07	0.07	0.26453
Stroop	0.59	0.136	0.17	0.04877
Count backwards	0.54	0.314	0.08	0.19927
Spot change	0.52	0.396	0.01	0.4807
Mental rotation	0.51	0.439	−0.01	0.54327
Map search	0.47	0.665	−0.01	0.52993
Indirect circle trace	0.36	0.97	−0.14	0.93302

Summary of AUC for classifications between fast-declining and stable subjects (extreme subgroups, subject numbers in Supplementary Table S5) using single tasks (permutation test). The Spearman rank correlation corresponds to the distance between the decision hyperplane and cognitive slopes using all pre-HD subjects from the test sites (within-site permutation test, see Methods).

**Table 3:** Model accuracies.

Task	AUC	p-value	Spearman’s rho	p-value
PC 1	0.67	0.013	0.18	0.04045
PC 2	0.59	0.11	0.06	0.2563
PC 3	0.56	0.23	0.09	0.19351
PC 4	0.73	0.0008	0.29	0.00306
PC 5	0.65	0.02	0.16	0.06194

Summary of AUC for classifications between fast-declining and stable subjects using principal components. The Spearman rank correlation corresponds to the distance between the decision hyperplane and cognitive slopes using all subjects from the test sites (within-site permutation test, see Methods).

**Figure 4:**
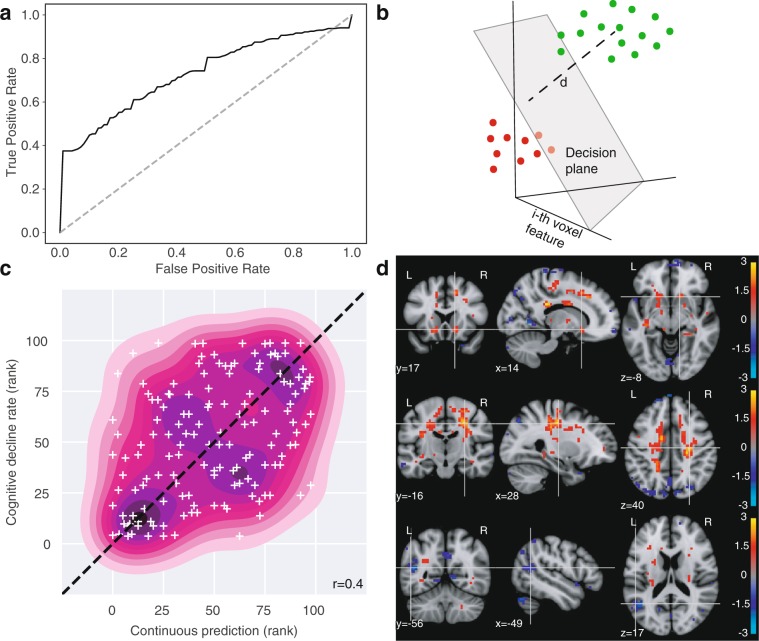
FCD stratifies pre-HD by longitudinal cognitive decline. (**a**) Classification of fast vs slow/stable subgroups of decline in SDMT task (healthy ageing/sex corrected) using full-brain FCD maps (LOSO-CV). Conventions as in Fig. 2b. Here ‘positive’ (vs. ‘negative’) labels denote ‘fast-declining’ (vs. ‘slow/stable’). AUC: 0.73 (p = 0.002, permutation test). (**b**) For linear classifiers, the distance to the decision plane provides a useful continuous output. (**c**) Relationship between FCD map distance to decision plane and behavioral slope of all pre-HD subjects, including the intermediate subgroup (scatter plot and 2-D kernel density estimation.). Average Spearman correlation: rho = 0.41 (p = 0.00005, permutation test). The distances to the decision hyperplane from each validation fold were converted to ranks to produce comparable units before pooling them together. Dotted line: identity line. (**d**) Voxelwise Spearman correlation between FCD and SDMT longitudinal performance decline reveal signatures in white matter. Top row: Bilateral clusters around head of the caudate/accumbens. Middle row: Large bilaterally symmetrical extensions of white matter extending from the striatum to motor cortex. Bottom: Left-temporal cortex FCD is anti-correlated with decline. (FDR at q < 0.05 in all panels). Units: logarithm of corrected p-value. Red: Higher in fast subjects. Blue: lower in fast subjects.

Binary classification of subjects from the extreme subgroups based on cognitive slopes from single tasks was possible for several tasks: SDMT, cancelation task, and paced tapping. The strongest results were observed for the SDMT task (Fig. 4). Visual inspection of the ROC curve (Fig. 4a, AUC: 0.73, p = 0.0023, permutation test) shows the predictive power resided in the correct identification of cases with highest confidence (vertical and horizontal segments near the extremes). The result suggests robust information in FCD maps about ongoing cognitive decline.

That validation tested whether brain patterns of ongoing decline could be learned, but not its application to the larger pre-HD population. However, the binary classifier can provide information in a setting where a patient might not belong to either subgroup but to the intermediate range not considered during training. This is because linear classifiers provide a continuous output: the (signed) distance to the decision hyperplane (Fig. 4b). To validate the usefulness of this measure in the whole pre-HD population, we computed on each test site the Spearman rank correlation between the distance to the decision hyperplane and the cognitive slope using all pre-HD subjects, and taking an average across sites (see Methods, “Validation of continuous classifier output for pre-HD stratification”). The average correlation was significant for the SDMT (Spearman’s rho = 0.41, p = 0.00005, permutation test) and the cancelation tasks (Spearman’s rho = 0.27, p = 0.00464, permutation test). This is illustrated for the SDMT task in Fig. 4c. We note that the out-of-sample correlations that can be achieved are limited by noise in cognitive slopes. As a reference, an optimistic estimate for the SDMT task (see Supplementary Information) indicates a ceiling for correlations at around 0.6. The results suggest the continuous output of the classification models can be informative for stratification by longitudinal decline of subjects from the whole pre-HD population.

What neurological signals underlie these predictions? We inspected the voxel-level correlations between FCD measures and SDMT slopes (Fig. 4d), which revealed patterns of association between SDMT decline and WM functional connectivity. These included clusters around the basal ganglia (Fig. 4d, top row), but mostly consisted of increased FCD on bilaterally-symmetrical WM extensions (Fig. 4d, center row), and also reduced FCD on cortical regions, notably left temporal cortex (Fig. 4d, bottom row). These observations reveal the spatial extent of signals related to early cognitive decline, and suggest WM as a source of functional signals associated with it in pre-HD.

The finding that stratification was possible for decline along more than one individual task could indicate the existence of multiple distinct decline signals, or simply one shared signal that underlies change along the individual tasks. We used the PCs of cognitive slopes to investigate whether stratification was possible along components of decline that were uncorrelated from each other, keeping in mind that because PCs are orthogonal by design they combine tasks with different signs, limiting interpretability. Stratification was possible, using the same LOSO-CV scheme, for three PCs (1, 4, and 5), in which models successfully distinguished extreme subgroups of decline (Table 3). The first component combined most tasks with positive signs, providing a summary of global decline. While the others are harder to interpret, we note PC 4 combined SDMT, cancelation, and self-paced tapping with the same sign, which were the tasks that showed best individual results. This suggests stratification along individual tasks might not be a consequence of a single dominant direction of decline, and instead stratification along multiple measures survives in decorrelated decline components.

### Robustness of FCD patterns to non-functional covariates

The goal of our analyses was not only to establish the predictive power of FCD maps but also to interpret them as reflecting functional signals. Therefore, even if predictions have been validated across imaging sites, we also verified the robustness of the identified patterns when controlling for non-functional sources of variation. In particular, we trained the main multivariate classification algorithms presented above (pre-HD vs. controls and stratification by SDMT decline) after correcting FCD features by a local atrophy measure (grey matter concentration as estimated by voxel-based morphometry, see Methods), removing variance attributable to scanning sites (even if there were no group differences on any of them, as assessed by chi-squared tests, see also Methods for details on harmonization), and skipping FCP map normalization by its median. Detection of pre-HD signatures (vs. controls) with these alternative FCD maps had similar predictive power (AUC: 0.64, p = 0.0005, permutation test), and the classifier weights showed a similar cluster in the globus pallidus (Supplementary Fig. S6). Performance of stratification by SDMT change was again comparable to the original model (AUC: 0.69, p = 0.007 permutation test; Spearman’s rho = 0.37, p = 0.00025, permutation test) and the voxel weights of the classifier revealed similar clusters (Supplementary Fig. S7) on white matter, while grey matter (GM) clusters on the temporal lobe were no longer observed, indicating perhaps concurrent GM atrophy. We also evaluated full-brain GMC maps (including WM voxels and with the same resolution as FCD maps), in terms of multivariate detection of pre-HD change (AUC 0.52, p = 0.378), and stratification by SDMT slopes (AUC 0.53, p = 0.39; Spearman’s r: 0.09, p = 0.24), showing low predictive power. Taken together, these observations suggest that the predictive patterns found are unlikely to be a reflection of local atrophy, site, or effects of normalization choice.

We also considered effects of in-scanner motion, as quantified by the mean framewise displacement (Methods, “Removal of motion associations”). Both groups had very similar motion distributions (pre-HD vs controls, p = 0.77, Mann-Whitney U-test, Supplementary Fig. S7). We computed motion associations with cognitive decline rates (Supplementary Table S7), which was significant for the SDMT (Spearman’s rho = −0.32, p = 0.006) and “spot the change” tasks (Spearman’s rho = 0.28, p = 0.02). Head motion association was not sufficient (e.g. “spot the change” task) nor necessary (e.g. cancelation task) for stratification results. We performed direct controls for FCD features in SDMT stratification (see Methods, “Removal of motion associations”). Both model performance and brain patterns were robust to this control (AUC: 0.73, p = 0.0009, permutation test; Spearman’s rho = 0.35, p = 0.00031, permutation test; Supplementary Fig. S9).

Finally, we considered whether addition of CAG repeats to the FCD features improved the stratification results. Results with these combined features were almost identical (Supplementary Table S8).

## Discussion

We investigated the reliability and stratification power of FCD in pre-HD using a cross-validated multivariate approach. FCD patterns showed high within-subject reliability. We found that longitudinal slopes provided a sensitive measure of early cognitive decline and FCD features could be used to stratify the population across multiple components of change. Together, these results highlight the potential of FCD as a tool with valuable applications.

Relative to structural markers, the literature of resting state measurements in HD is relatively recent^14^. Cross-sectional correlations of resting-state fMRI with clinical measures were reported on predefined components^47,48^. With that approach, cross-sectional group differences were observed^49^ within the TRACK-HD study but longitudinal group-differences were not^50^. The Track-On HD extension allowed for the investigation of putative compensation mechanisms^20^ and relationships with the anatomical connectome^24^. A valuable step toward predictive applications has been a recent study combining structural and resting-state markers in 19 premanifest patients used to predict transition to the manifest state^51^. Our goal was to further push the field from descriptive brain mapping toward predictive models that directly detect the population heterogeneity unexplained by genetic disease load, before manifestation.

Key for the usefulness of fMRI as a tool is verifying its robustness across known sources of variability such as sites and individuals. Site heterogeneity has been suggested to be a major challenge for robustness of models, and of LOSO-CV has been proposed as a methodology to ensure generalizability^12^. This was the motivation behind our choice. However, it is possible that the efforts made to minimize site discrepancies during acquisition in Track-ON^20^ make site differences unrepresentative of those found in other potential practical applications. In terms of within-subject consistency, concerns have been raised regarding the reliability of fMRI for biomarkers to be applied at the individual level^52^. The use of multivariate models avoid the important consistency issues of single links: models that rely on global patterns have been shown to be robust enough to enable individual fingerprinting^11^. This allowed us to extend assessments from other studies of test-retest reliability for rs-fMRI in pre-HD^53^ to the individual level. We found that FCD maps contained a highly reproducible signature that identified individuals. This extends robustness to a new feature type, FCD, but also in time, since the scans considered were separated by one or two years. More important neurologically, we found that the mild pre-HD signatures are highly reproducible within-subjects (0.95 consistency in pre-HD subjects), highlighting the importance of sorting out the reproducible heterogeneity.

The single clearest signature of pre-HD in FCD was bilateral hyper-connectivity centred on the globus pallidus and its interface with the putamen. This is striking because the globus pallidus is not prominent in markers of volume change compared to the putamen. Anomalous FCD of the globus pallidus in the past was perhaps previously unobserved because of the use of predefined components^47^ or networks^20^ that excluded this region. It was unperturbed when correcting by GMC. In fact, the same models using full-brain GMC maps did not significantly distinguish the groups. We note that GMC could distinguish groups with 70% accuracy in the larger TRACK-HD cohort^40^ in models that included only grey matter voxels, higher resolution voxels, and a different cross-validation scheme. Even then, the globus pallidus did not contribute to that discrimination. Interestingly also, the pallidus pattern seemed to be a generic signature of pre-HD, but did not appear to be relevant for the cognitive decline stratifications within the pre-HD population.

In contrast, the recurrent finding in our stratification by cognitive decline is the hyper-connectivity of white matter (WM). Although until recently disregarded as noise, there is no evidence against the detection of physiological BOLD signals in WM^54^. On the contrary, several studies have found reproducible WM activations in tasks requiring interhemispheric integration^55–58^, and several features that speak to their physiological origin: they depend on diffusion anisotropy and fibre orientation^59^, on activity of connected cortical areas^18,19,29^, and alertness^28^. In resting state fMRI, highly reproducible WM networks have been reported and are associated with combinations of known tracts^18^. Of particular interest, WM activations were reported in SDMT specifically^60^, the task where we found the largest WM associations. WM signatures have started only recently to be exploited as potential markers of brain disease^16^. This is the first demonstration of functional WM signals in HD. Future studies should focus on the mechanisms of white matter functional signals in neural degeneration.

We found SDMT to be more strongly associated with FCD features than any other measure, either individual tasks or PCs. SDMT has long been recognized as one of the best measures of early deterioration in HD^5,61^. In this dataset, it is the task that showed strongest group differences in longitudinal decline. It is possible that this sensitivity of the task to tracking progression, in combination with reliance on white matter integrity^62,63^, explains its advantage over the other tasks considered. PCs were meant to isolate different dimensions of cognitive change in an unsupervised way. In addition, they are orthogonal by design, and as a result of this constraint can mix tasks sensitive to disease progression with those that are not. These properties of the method could explain why a high-sensitivity task could have a stratification advantage over PCs.

The interpretation of the current results should not be considered separately from the limitations of this study. First, the cohort was restricted to premanifest participants, which limited our conclusions to the premanifest period and likely introduced a selection bias, as suggested by the strong anticorrelation between age and CAG repeats. Similarly, inclusion of only right-handed subjects, common in the neuroscientific literature, can limit clinical applications. In addition, cross-validated analyses could have overoptimistic results, particularly if care is not taken to exclude the possibility of “double-dipping”, as in the choice of model hyperparameters^64^. While we made efforts to avoid such instances using nested-cross-validation^65^, conclusions should be validated on a separate, independent cohort. In terms of cognitive/motor changes, available data included visits that preceded rs-fMRI acquisition for the sake of maximizing power to detect changes. Because of that, one should not interpret our models as evidence of the ability to predict future decline but rather to detect ongoing cognitive change; and prediction in this context refers to the ability to make assessments for individuals not considered during model training. Similarly, AUC-based validations were based on binary labels that did not include the full pre-HD population and should not be taken as evidence of usefulness in more general clinical contexts. Correlations with continuous decline using the full population (our second validation) should be considered as effect sizes for that scenario. Finally, the signatures found by our models are partly a consequence of the type of model chosen. Multiple model types are possible, with different complexities. However, this is an issue for every analysis effort, even univariate comparisons, which assume a type of model implicitly. We used a linear model for its simplicity of interpretation.

**Figure 5:**
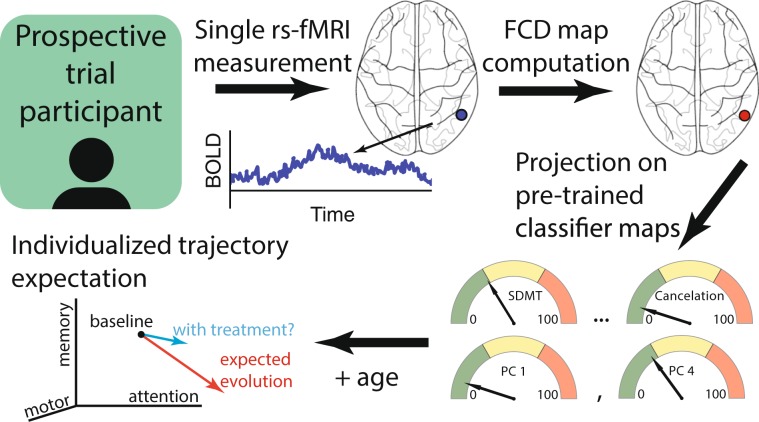
Hypothetical application of FCD information about cognitive/motor change to individuals. A single fMRI measurement would be obtained on an individual. The corresponding FCD map would be projected onto pre-trained classifier weights which would help position the patient by expected decline along single cognitive/motor measures or combinations of them (similar to Fig. 4c). Patients in which ongoing change is expected could receive treatment and their evolution could be compared against an expected individualized trajectory, increasing sensitivity.

Early progression of HD is usually modelled in terms of the CAP score^5,66^ and predictive models have used this, sometimes combined with cognitive^61^ and brain measurements^51,67^, for the prediction of the age of diagnosis or conversion^68,69^. Part of the value of our approach lies in its acceptance that degeneration trajectories are heterogeneous, placing individuals in a multidimensional space. Accordingly, we focused on the prediction of longitudinal change as opposed to estimating proximity to a given behavioural threshold, as schematized in Fig. 5. Such multidimensional estimates could not only inform patient selection in clinical trials, but potentially be used for individualized quantification of treatment effect sizes and expected patient evolution from ongoing decline, increasing sensitivity.

In conclusion, we found robust evidence for the usefulness of FCD as a stratification tool. This is critical because subjects showing signs of decline are unlikely to slow their course in the absence intervention. In this context, FCD provides a new window into the evaluation, in a single visit, of ongoing change, an important type of assessment for which few alternatives exist.

## Supplementary information

Supplementary Information.

## Data Availability

For the privacy of gene carriers and control participants who generously provided data for the Track-On HD study, the dataset is not posted online for direct download. The original unprocessed TRACK-IDS-2015-10-R2.1 dataset will be made available upon request after appropriate data use agreements are signed. Please direct inquiries to info@chdifoundation.org with the words [IBM-CHDI Resting State fMRI Study] in the subject line.

## Code availability

All scripts used for generating the results of this study are publicly available as a Github repository (https://github.com/polosecki/ton_rfmri_repo) for reproducibility.
