# Supplementary material for: Resting-state connectivity stratifies premanifest Huntington’s disease by longitudinal cognitive decline rate
Source: Sci Rep. 2020 Jan 27;10:1252. doi: 10.1038/s41598-020-58074-8 (PMC6985137; doi:10.1038/s41598-020-58074-8)
Supplement: Supplementary file 1 — Supplementary Information. [file 41598_2020_58074_MOESM1_ESM.pdf]

Supplementary Information for "Resting-state connectivity stratifies premanifest Huntington's disease by longitudinal cognitive decline rate"

Pablo Polosecki[1], Eduardo Castro[1], Irina Rish[1], Dorian Pustina[2], John H. Warner[2], Andrew Wood[2],

Cristina Sampaio[2] and Guillermo A. Cecchi[1]

[1]IBM T.J. Watson Research Center, Yorktown Heights, NY, USA
[2]**CHDI Management/CHDI Foundation, Princeton, NJ, USA**

Brief description of cognitive/motor tasks

Stroop Test: Subjects must read as many words as possible in 45 seconds from a list of the names of colors printed in black ink (reading condition). **Measure**: number of words read correctly.

Symbol Digit Modalities Test (SDMT): Subjects are given 90 seconds to match symbols and digits as quickly as possible by writing, using a key at the top of the page. **Measure**: number of correct responses.

Paced Tapping: Initially a tone is presented at a constant rate (3Hz). Subjects begin by tapping with alternating thumbs at the same rate as the tone. After 12 taps the tone is discontinued and subjects attempt to maintain the timing for 31 taps. 5 such trials take place. **Measure**: logarithm of the standard deviation of inter-tap intervals.

Circle Tracing: Subjects trace a 90mm-diameter circle as quickly and accurately as possible, aiming to stay within a 5mm annulus. Their hand and drawing tablet are hidden from view and visual feedback is presented on a vertically-placed monitor (indirect condition). **Measure**: length traced within the annulus in 45 seconds.

Map Search: Subjects must search and mark a target symbol occurring on several locations on a map among other distracter items. **Measure**: symbols found in one minute.

Cancellation: Subjects must locate stimuli defined by combinations of visual features randomly distributed among distractors. **Measure**: number of correct identifications in 90 seconds.

Spot the Change: Subjects are briefly (0.25s) presented with an array of five colored squares. After a pause of 1s, they are presented with a similar array where one of the locations is highlighted. Subjects must report if the square in the highlighted location has changed. **Measure**: Cowan's K formula for estimating the number of items encoded.

Mental Rotation: Subjects are presented with two 3-D shapes and must indicate if one is a rotation of the other or that of a mirror image instead. **Measure**: % correct.

Counting Backwards: Subjects must count backwards by a given step size while they perform the circle tracing task. **Measure**: Number of correct counts.

Grip force: Subjects must hold a 500g object with their dominant hand while their gripping force is recorded. **Measure**: temporal standard deviation of the griping force.

Detailed descriptions are found in the original study by Klöppel and colleagues[1].

Estimation of noise in cognitive slopes

The signal-to-noise relationship in cognitive slopes is an important issue, but it is not frequently discussed in the literature. Here we make an effort to estimate it for the task where we observe the strongest brain signals: the SDMT task. Slope noise depends on measurement noise at each time point and the number of them used for slope estimation. Slope signal depends on the average decline relative to the control group plus its heterogeneity within the clinical population. In addition, it is proportional to the duration of the time window used for computing it.

Here, we estimate measurement noise at each time point from estimates of test-retest reliability of the SDMT task in the literature. Test-retest reliability is determined by noise measurement and the overall performance heterogeneity in the population. The one study of SDMT reliability in HD combined pre-HD, manifest HD and control population in its estimation, greatly increasing the heterogeneity signal relative to what would be expected in a pre-HD population[2]. It estimated a reliability of 0.93. It has been more thoroughly studied in other clinical populations such as multiple sclerosis, where reports of reliability oscillate around 0.9[3]. We take this value here, considering it a likely optimistic value for pre-HD given the population heterogeneity in the study of Stout an colleagues[2]. It should be noted that final slope noise estimates are sensitive to this parameter, and future measurements of it are of important for the detection of cognitive change in specific populations.

Regarding total baseline variability, standard deviation of baseline measurements in the pre-HD population is 10.3[4]. This allows estimation of measurement noise as:

$\sigma_{noise} = \sigma_{total}\sqrt{(1 - r_{test-retest}{}^2)} = 4.5$ symbols

Using this estimate of measurement noise, we can simulate a slope noise distribution, given a number N_{visits} of visits, by fitting a slope to N_{visits} samples taken from a normal distribution with standard deviation σ_{noise}. In this study subjects did not all have the same number of N_{visits}, so we combined different values of N_{visits} in the proportion given for the SDMT task in Figure S1. The resulting noise distribution is shown in Figure S8. It is a mixture of gaussians with different widths. Its variance is our estimate of slope noise variance var_{noise}.

From the empirical distribution of observed slopes in the pre-HD population (Figure S10), we computed the mean squared deviation from the healthy slope average, which we called var_{total}. This includes deviations due to true neurological heterogeneity plus noise:

$var_{total} = var_{neurological} + var_{noise}$

From which we get the fraction of the variance accounted by neurological signal:

$\frac{var_{neurological}}{var_{total}} = \frac{var_{total} - var_{noise}}{var_{total}}$

Plugging in our simulated estimate of noise and observed variability in slopes, the neurological variability is approximately 37% of the total. This puts a ceiling on the correlation one could obtain with a predictive model at approximately 0.6. While this is a rough estimate, with several assumptions, it motivates the use of coarse-grained labels as opposed to fine-grained estimations of decline from continuous values.

Supplementary References

1. Klöppel, S. *et al.* Compensation in Preclinical Huntington's Disease: Evidence From the Track-On HD Study. *EBioMedicine* **2,** 1420–1429 (2015).
2. Stout, J. C. *et al.* HD-CAB: a cognitive assessment battery for clinical trials in Huntington's disease 1,2,3. *Mov. Disord.* **29,** 1281–1288 (2014).
3. Benedict, R. H. B. *et al.* Reliability and equivalence of alternate forms for the Symbol Digit Modalities Test: implications for multiple sclerosis clinical trials. *Mult. Scler.* **18,** 1320–1325 (2012).
4. Stout, J. C. *et al.* Evaluation of longitudinal 12 and 24 month cognitive outcomes in premanifest and early Huntington's disease. *J. Neurol. Neurosurg. Psychiatr.* **83,** 687–694 (2012).

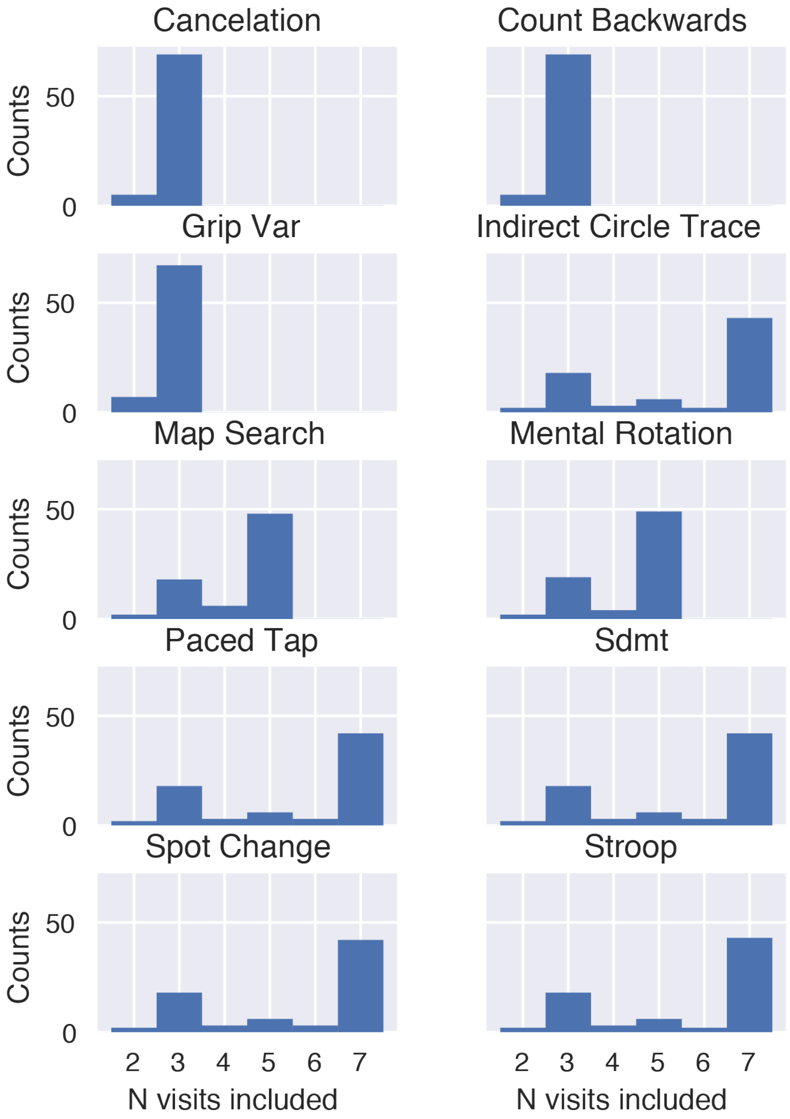

Figure S1 – Distribution of visits available for the computation of cognitive slopes on each task in pre-HD subjects. The peaks at N=7 and N=3 correspond to subjects with all TRACK-HD/TRACK-ON and TRACK-ON visits respectively.

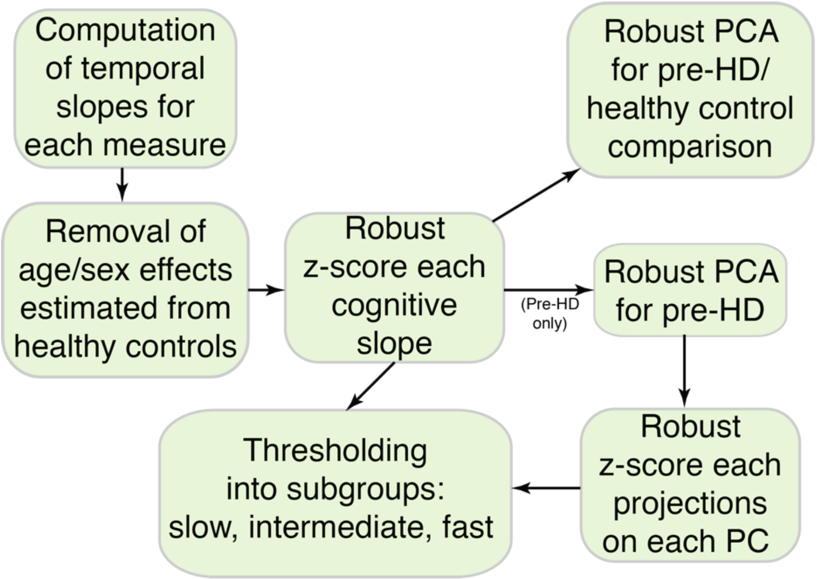

Figure S2 – Schematics of the assignment of subjects to subgroups of cognitive change. In brief, after removal of expectations from healthy age and sex either single tasks or principal components of uncorrelated change were used for dividing the population into three subgroups.

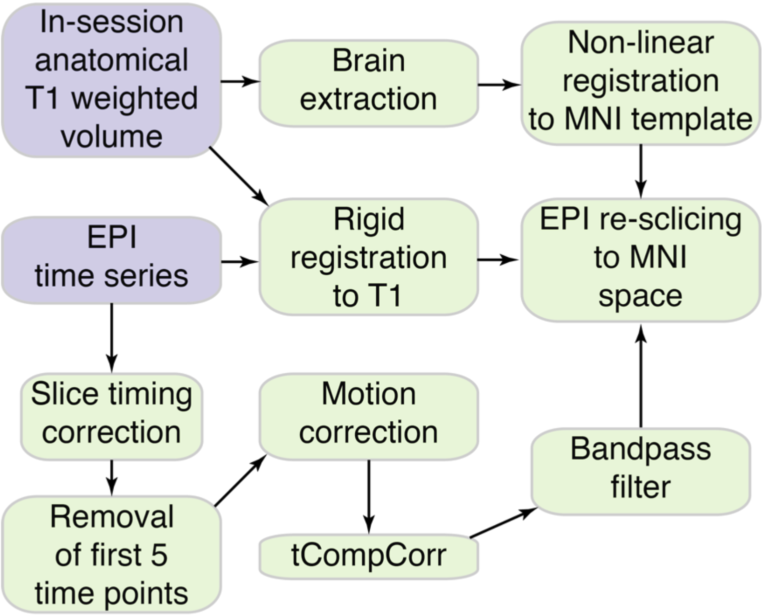

Figure S3 – Schematics of fMRI preprocessing pipeline. Standard steps were followed including slice-timing correction, removal of first time points to keep steady-state scans, motion correction by rigid realignment to middle time point, tCompCorr removal of physiological and motion signals, and band-pass filter of slow drifts and high-frequency noise. Finally, the time series was non-linearly transformed to MNI coordinate space.

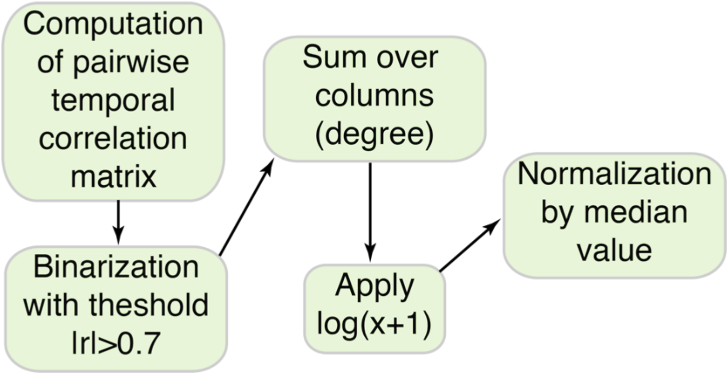

Figure S4 – Schematics of FCD map computation. A voxel level pairwise correlation matrix was computed, and binarized at an absolute value of 0.7. We computed the degree centrality of each voxel by summing over columns. We then computed the natural logarithm of degree (plus one) and normalized by the global median.

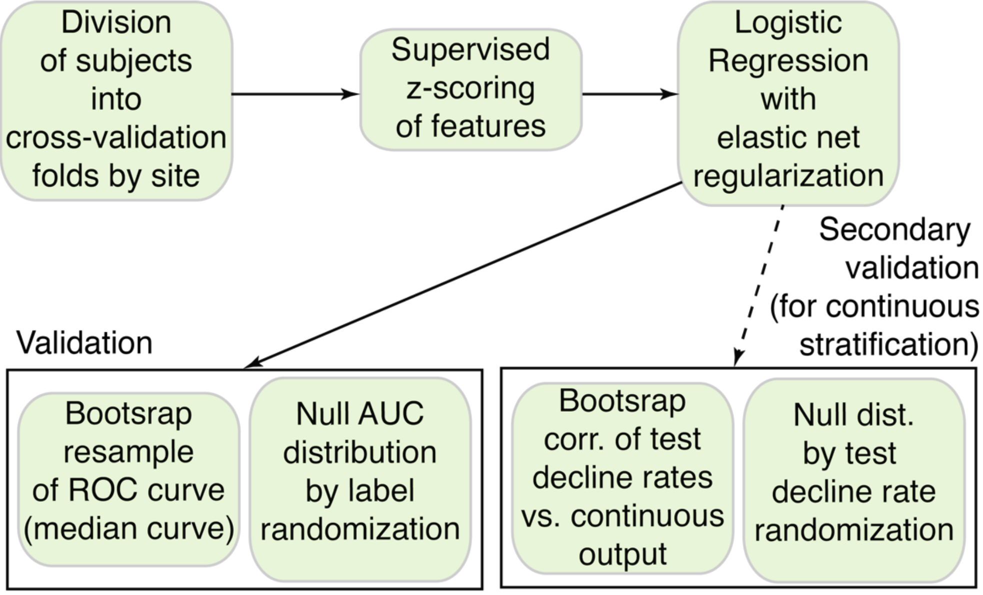

Figure S5 – Schematics of classification pipeline. After test subjects were removed from training, each feature was z-scored, and a logistic regression classifier with elastic net regularization was fitted. In stratification models, training was performed including fast and slow decline subgroups only (binary classification). For test subjects, FCD features were z-scored using the coefficients learned during training. We produced an ROC curve for each cross-validation fold and averaged them across folds. In stratification models, ROC curves quantified ability to distinguish the extreme fast and slow subgroups in the test sites. For increased robustness, the ROC was resampled using the bootstrap (random resampling of test subjects with replacement, 10^5 resamples) and the median ROC was obtained, along with the median ROC AUC. A null distribution was obtained by repeating the AUC calculation after permutation of test labels within each test site (10^5 permutations). In addition, for stratification models, we quantified the predictive power of the continuous distance to the decision hyperplane by computing its correlation with the continuous cognitive decline rate including all pre-HD subjects on each test site. A null distribution was obtained by repeating the correlation calculation after permutation of decline rates within each test site (10^5 permutations).

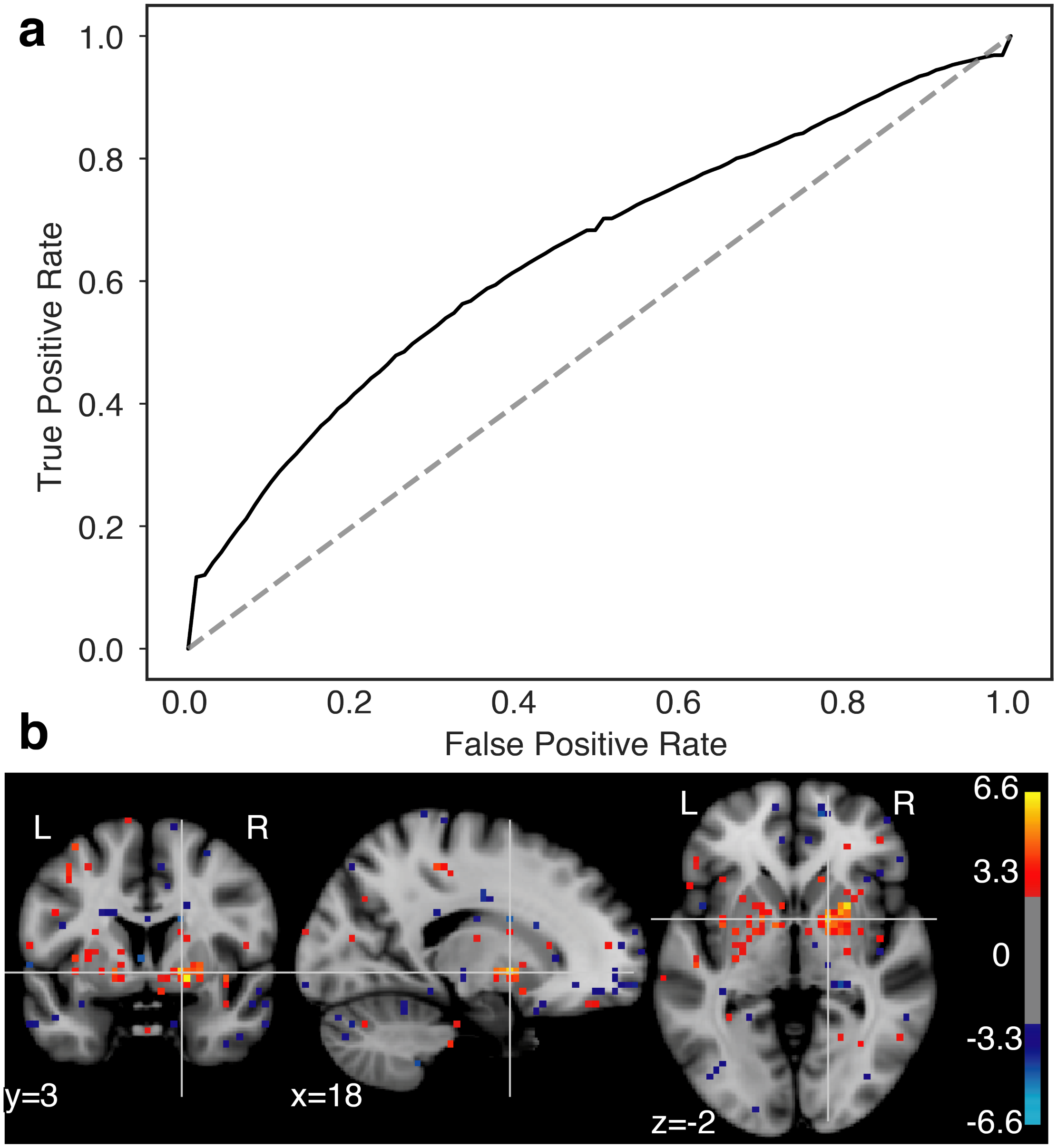

Figure S6 – Robustness of globus pallidus pre-HD FCD patterns. (**a**) Similar to Figure 2b, controlling for local atrophy, site effects, and omitting normalization by the median. AUC: 0.64 (p=0.0005, permutation test) (**b**) Similar to **Figure 2e**, for these controlled features.

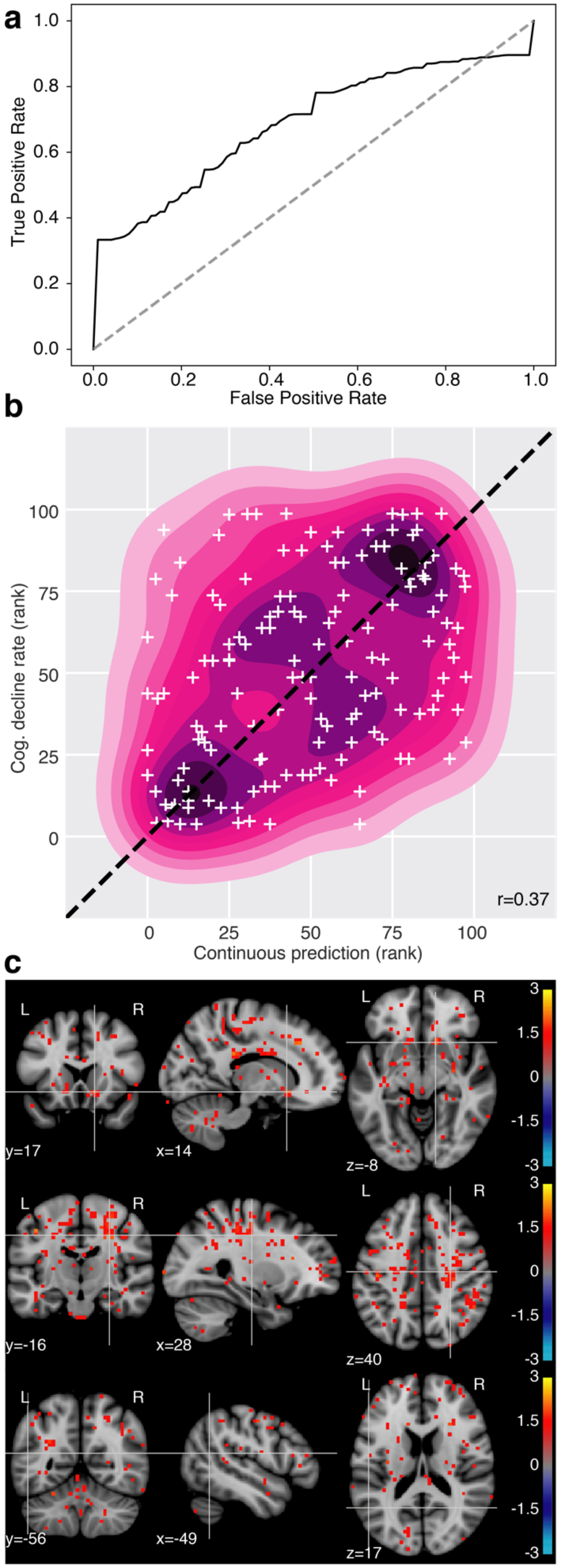
a
1.0
0.8
0.6
0.4
0.2
0.0
True Positive Rate
0.0
0.2
0.4
0.6
0.8
1.0
False Positive Rate
b
100
75
50
25
0
Cog. decline rate (rank)
0
25
50
75
100
Continuous prediction (rank)
r=0.37
c
L
R
y=17
x=14
z=-8
y=-16
x=28
z=40
y=-56
x=-49
z=17
3
1.5
0
-1.5
-3

Figure S7 – Robustness of stratification patterns. (**a**) Similar to **Figure 4a (a copy of Fig. 4 is provided on p. 17 for convenience)**, controlling for local atrophy, site effects, and omitting normalization by the median. AUC: 0.69 (p=0.0075, permutation test). (b) Similar to **Figure 4b**, for these controlled features. Spearman correlation: 0.37, p=0.00025, permutation test. (**c**) Similar to **Figure 4d**, for these controlled features. The left STS cortical connectivity reduction is not present in these features, suggesting concurrent local grey matter density changes.

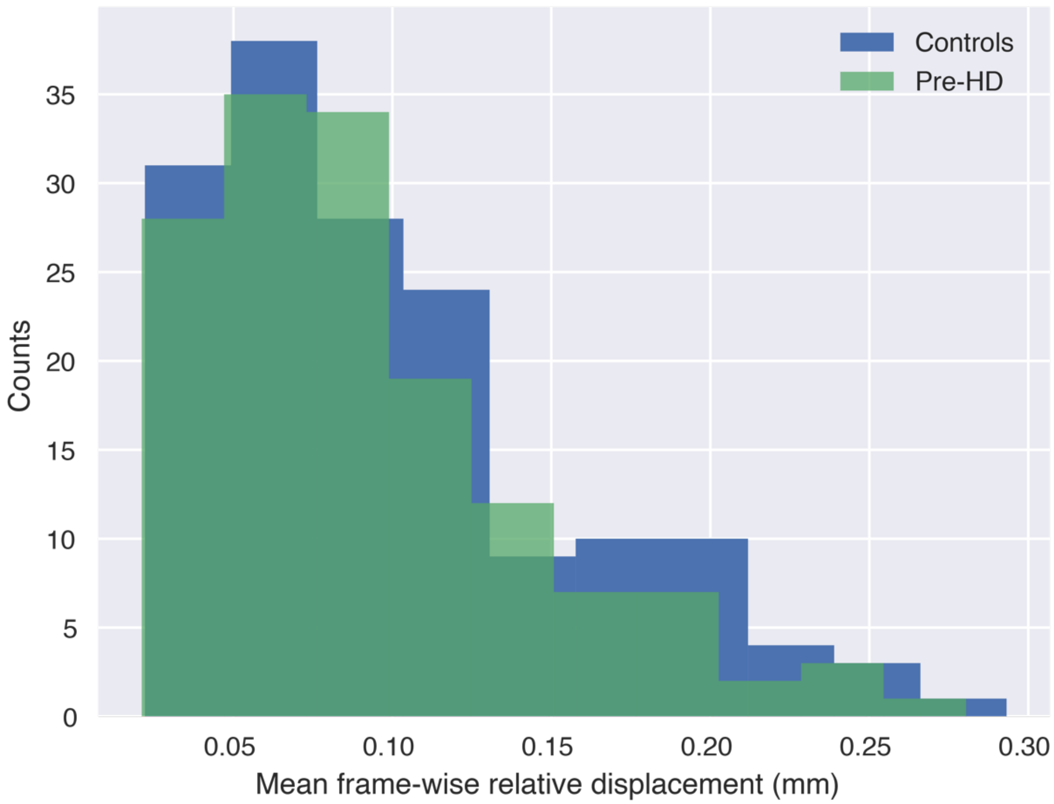

Figure S8 – Distribution of head motion in pre-HD and control subjects. The mean frame-wise displacement was similarly distributed between the two groups (p=0.77, Mann-Whitney U-test).

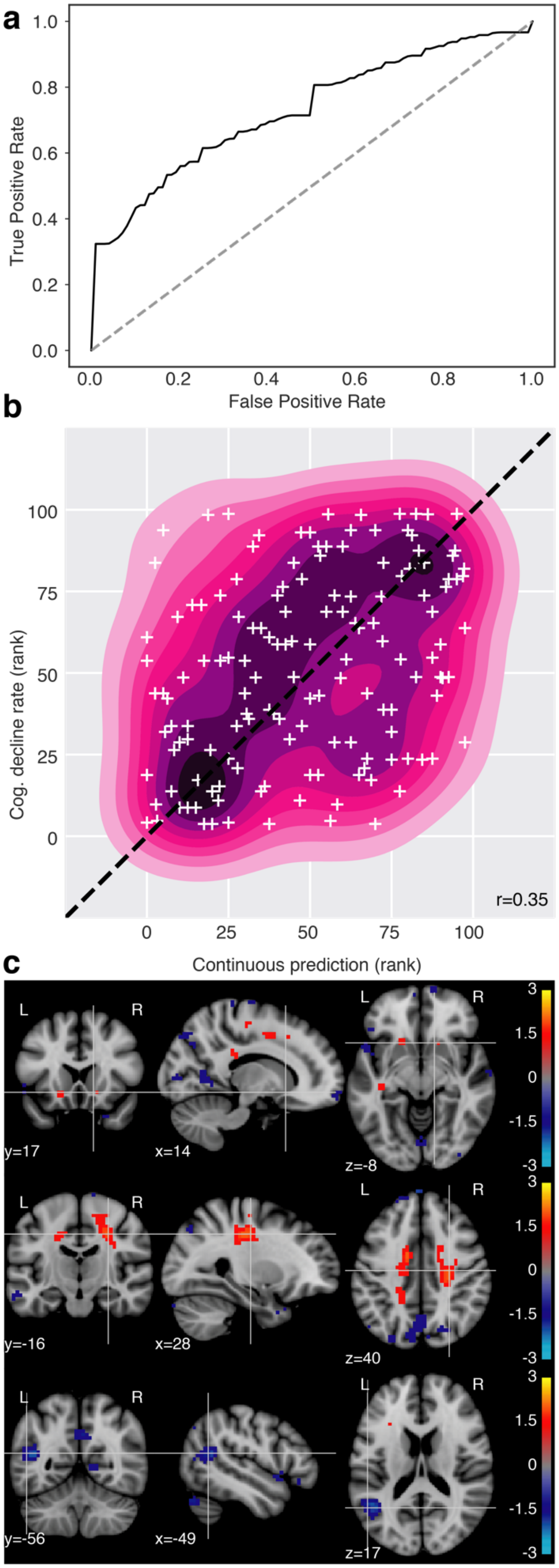
a
True Positive Rate
False Positive Rate
b
Cog. decline rate (rank)
Continuous prediction (rank)
r=0.35
c
L
R
y=17
x=14
z=-8
y=-16
x=28
z=40
y=-56
x=-49
z=17

Figure S9 – Robustness of stratification patterns. (**a**) Similar to **Figure 4a (a copy of Fig. 4 is provided on p. 17 for convenience)**, controlling for head motion (mean frame-wise displacement). AUC: 0.73 (p=0.00089, permutation test). (b) Similar to **Figure 4b**, for these controlled features. Spearman Correlation: 0.35, p=0.00031, permutation test. (**c**) Similar to **Figure 4d**, for these controlled features.

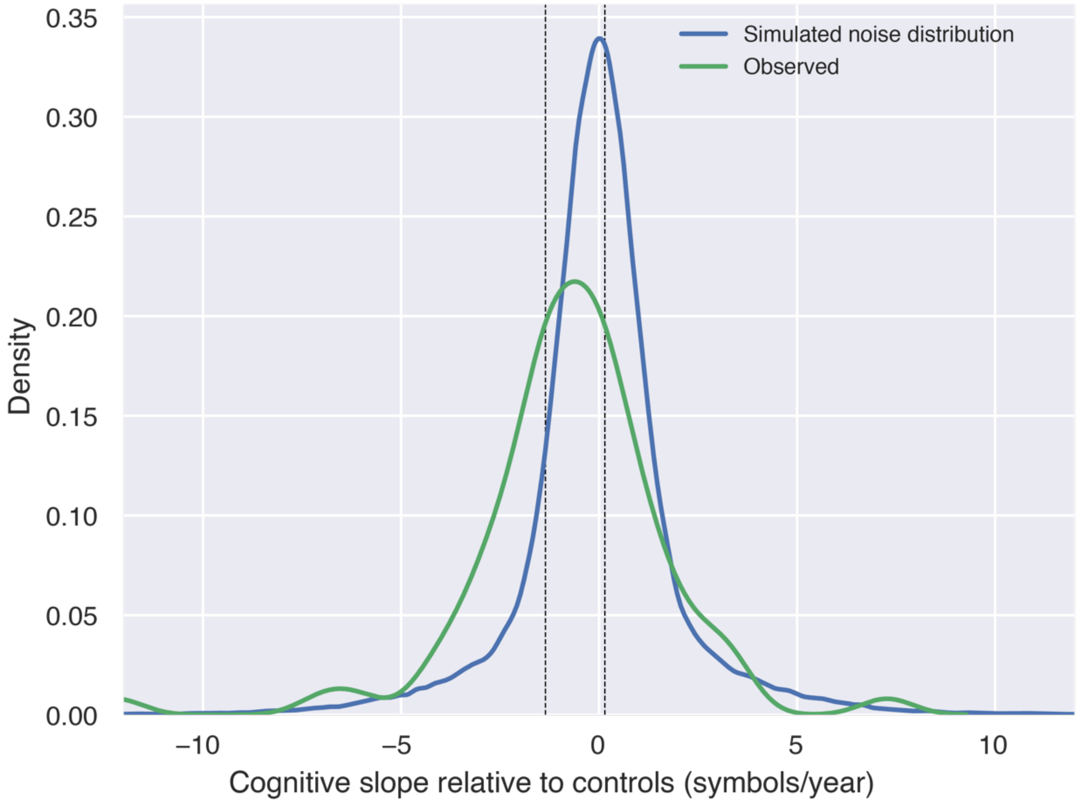

Figure S10 – Estimation of noise distribution in SDMT slopes. Kernel density estimates of simulated noise distributions (blue) and observed SDMT slopes (green) in pre-HD subjects (see Supplementary text). In this estimation true decline accounts for 37% of the observed variance from control subjects, posing a ceiling to predictive correlations around 0.6.

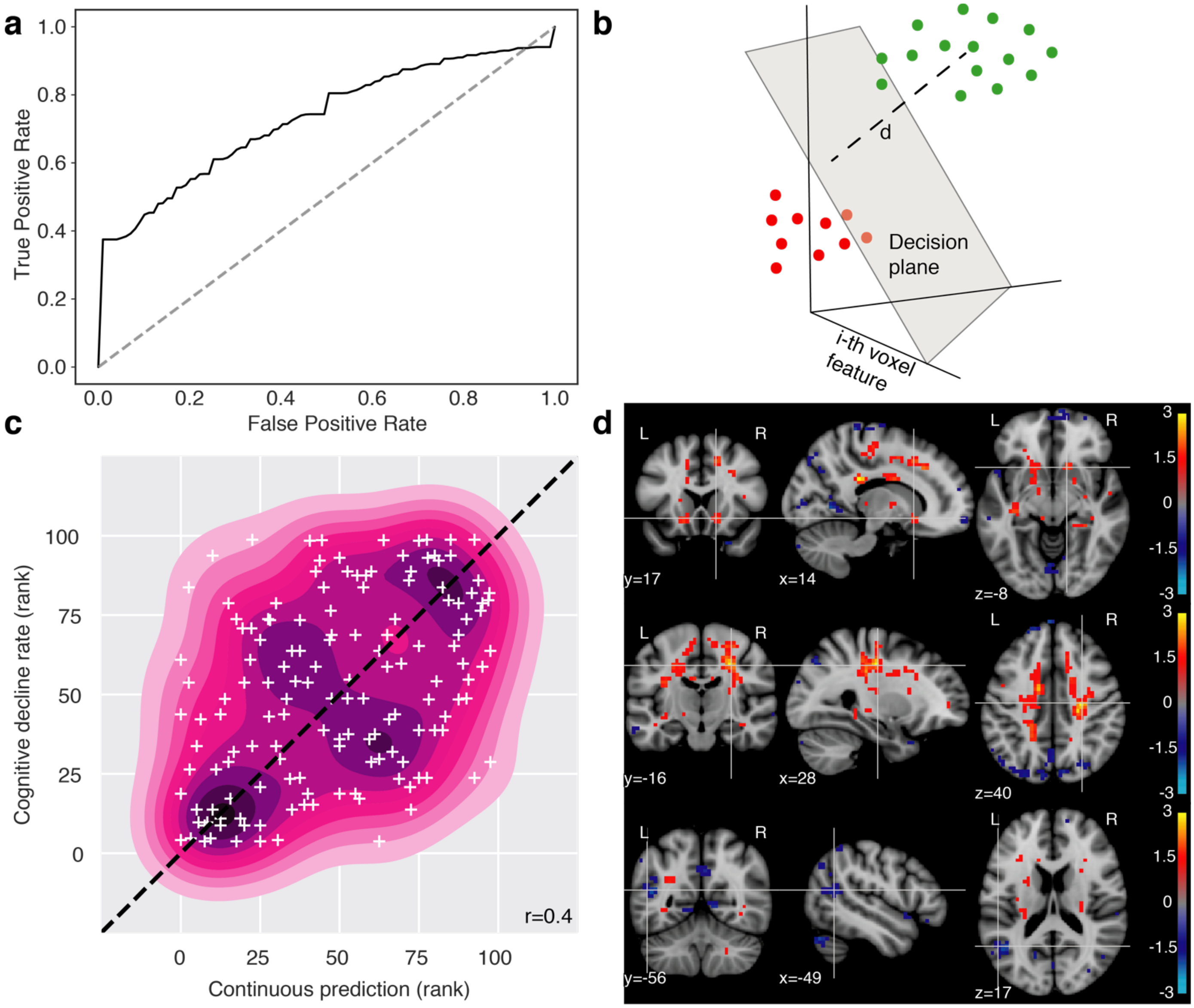

Figure 4 (copy of Fig. 4 from main text provided to facilitate comparison with control analyses presented here) - FCD stratifies pre-HD by longitudinal cognitive decline. (**a**) Classification of fast vs slow/stable subgroups of decline in SDMT task (healthy age/sex corrected) using full-brain FCD maps (LOSO-CV). Conventions as in **Figure 2B**. Here 'positive' (vs. 'negative') labels denote 'fast-declining' (vs. 'slow/stable'). AUC: 0.73 (p=0.002, permutation test) (**b**) For linear classifiers, the distance to the decision plane provides a useful continuous output. (**c**) Relationship between FCD map distance to decision plane and behavioral slope of **all** pre-HD subjects, including the intermediate subgroup (scatter plot and 2-D kernel density estimation.). Average Spearman correlation: rho=0.41 (p=0.00005, permutation test). The distances to the decision hyperplane from each validation fold were converted to ranks to produce comparable units before pooling them together. Dotted line: identity line. (**d**) Voxelwise Spearman correlation between FCD and SDMT performance longitudinal decline reveal signatures in white matter. Top row: Bilateral clusters around head of the caudate / accumbens. Middle row: Large bilaterally symmetrical extensions of white matter extending from the striatum to motor cortex. Bottom: Left-temporal cortex FCD is anti-correlated with decline. (FDR at q < 0.05 in all panels). Units: logarithm of corrected p-value. Red: Higher in fast subjects. Blue: lower in fast subjects.

	Lost	Remaining
Registered in study	-	245
With some imaging file	4	241
At least 2 rs-fMRI sessions	16	225
Successful Preprocessing	4	221
Low motion	4	217
Not converted	10	207
Righthanded	22	185
All task slopes	14	171
Successful anatomical preprocessing	18	153

Table S1 – Excluded subjects. List of issues that cause exclusion of different subjects, from all subjects registered in the database to the 153 used in this analysis.

Task	Mann-Whitney Statistic	p-value
SDMT	3566	0.009
Cancelation	2727	0.763
Spot change	3355	0.058
Stroop	3507	0.017
Indirect circle trace	3432	0.032
Paced tap	3522	0.014
Map search	2866	0.583
Count backwards	3288	0.092
Grip variability	2882	0.560
Mental rotation	2943	0.472

Table S2 – Group differences in cognitive decline slopes. Statistical tests of greater decline in the pre-HD group relative to healthy controls (one-sided Mann-Whitney U Test).

Task	Healthy aging		CAG repeats		CAP score	
	Spearman's rho	p-value	Spearman's rho	p-value	Spearman's rho	p-value
SDMT	-0.10	0.40	0.00	0.97	-0.12	0.32
Cancelation	0.11	0.32	-0.04	0.71	-0.08	0.49
Spot change	-0.21	0.06	-0.14	0.25	-0.14	0.22
Stroop	0.03	0.78	-0.20	0.09	-0.25	0.03
Indirect circle trace	-0.04	0.71	-0.05	0.65	-0.13	0.26
Paced tap	0.08	0.50	0.04	0.75	-0.06	0.60
Map search	-0.03	0.81	0.08	0.51	-0.02	0.89
Count backwards	0.16	0.17	-0.22	0.06	-0.30	0.01
Grip variability	-0.01	0.92	-0.14	0.25	-0.10	0.41
Mental rotation	-0.11	0.34	-0.07	0.55	-0.25	0.03

Table S3 – Decline associations. Spearman's rank correlation between task slopes and aging (in controls), CAG repeats, and CAP score (in pre-HD).

Task	Spearman's r	p-value
Cancelation	0.19	0.10
Count backwards	0.23	0.05
Grip variability	0.01	0.92
Indirect circle trace	-0.18	0.13
Map search	0.21	0.07
Mental rotation	-0.20	0.08
Paced tap	-0.21	0.08
SDMT	0.17	0.16
Spot change	-0.18	0.12
Stroop	-0.05	0.68

Table S4 – Correlation between cognitive slopes and number of visits in pre-HD subjects.

	Subject count			Age [mean (sd)]			CAG [mean (sd)]			CAP [mean (sd)]		
	Slow	Interm.	Fast	Slow	Inter,	Fast	Slow	Interm.	Fast	Slow	Interm.	Fast
PC 1	23	27	24	45 (10)	41 (10)	40 (9)	42.2 (1.6)	43.4 (2.9)	43.7 (2.2)	46 (6)	49 (11)	49 (7)
PC 2	24	27	23	42 (10)	44 (10)	40 (9)	43..1 (2.3)	42.5 (2.2)	43.8 (2.5)	48 (8)	47 (8)	50 (9)
PC 3	26	26	22	42 (11)	42 (9)	41 (10)	43.3 (3.0)	42.6 (1.3)	43.5 (2.5)	49 (10)	46 (6)	50 (8)
PC 4	24	29	21	47 (11)	42 (8)	37 (7)	42.3 (2.3)	43.1 (2.1)	44.1 (2.5)	48 (8)	49 (9)	48 (7)
PC 5	26	24	24	39 (8)	43 (10)	44 (11)	43.9 (2.3)	42.8 (2.1)	42.7 (2.6)	51 (9)	47 (8)	47 (8)
Cancelation	21	28	25	40 (11)	43 (8)	43 (11)	43.7 (2.8)	43.1 (2.0)	42.7 (2.4)	49 (8)	50 (8)	46 (8)
SDMT	19	30	25	40 (9)	44 (9)	42 (11)	43.2 (2.0)	43.0 (2.3)	43.3 (2.8)	46 (4)	50 (9)	48 (9)
Grip variability	25	26	23	41 (8)	44 (11)	40 (10)	43.1 (2.2)	42.7 (2.6)	43.7 (2.2)	48 (8)	47 (8)	50 (8)
Stroop	23	30	21	40 (9)	46 (9)	39 (9)	43.2 (2.5)	42.6 (2.2)	43.8 (2.4)	46 (7)	50 (9)	48 (8)
Map search	17	30	27	44 (12)	40 (8)	43 (10)	43.0 (2.9)	43.5 (1.9)	42.8 (2.5)	48 (9)	49 (8)	47 (8)
Spot the change	24	26	24	47 (10)	41 (8)	37 (8)	41.8 (1.7)	43.3 (2.4)	44.3 (2.3)	46 (8)	49 (9)	50 (8)
Mental rotation	23	29	22	41 (10)	43 (10)	41 (9)	43.0 (2.8)	42.9 (2.0)	43.5 (2.4)	46 (9)	49 (7)	50 (9)
Count back.	22	28	24	42 (10)	44 (9)	40 (9)	42.8 (2.5)	42.4 (1.7)	44.3 (2.5)	46 (8)	46 (8)	52 (8)
Paced tap	23	31	20	41 (9)	42 (10)	43 (11)	43.1 (2.3)	43.0 (2.6)	43.3 (2.2)	48 (9)	47 (9)	50 (6)

Table S5 – Subjects assigned to each decline subgroup by cognitive measure, and subgroup demographics.

Task	Mann-Whitney Statistic	p-value
SDMT	264	0.51
Cancelation	304.5	0.06
Spot change	296.5	0.86
Stroop	230.5	0.79
Indirect circle trace	198	0.21
Paced tap	196.5	0.38
Map search	278	0.19
Count backwards	296	0.20
Grip var	302	0.59
Mental rotation	199.5	0.17

Table S6 – Difference in number of visits on extreme decline subgroups used for training. Mann-Whitney U Test.

Task	Spearman's r	p-value
SDMT	-0.319	0.006
cancelation	-0.016	0.895
Spot change	0.280	0.016
Stroop	-0.053	0.653
Indirect circle trace	0.121	0.304
Paced tap	-0.204	0.082
Map search	-0.160	0.173
Count backwards	-0.039	0.741
Grip var	-0.110	0.352
Mental rotation	0.138	0.242

Table S7 – Relationship between cognitive decline rates and mean framewise displacement.

Cognitive measure	AUC	p-value	Spearman's r	p-value\
PC 1	0.67	0.013	0.19	0.028
PC 2	0.58	0.13	0.05	0.31
PC 3	0.51	0.45	0.08	0.22
PC 4	0.72	0.0012	0.28	0.0034
PC 5	0.57	0.21	0.16	0.062
SDMT	0.73	0.0021	0.41	0.00006
Cancelation	0.72	0.0028	0.27	0.0043
Paced tap	0.68	0.02	0.07	0.26
Grip variability	0.61	0.08	0.07	0.27
Stroop	0.59	0.14	0.16	0.055
Count backwards	0.55	0.27	0.07	0.22
Spot change	0.52	0.39	0.00	0.49
Mental rotation	0.50	0.48	0.00	0.51
Map search	0.46	0.70	0.00	0.51
Indirect circle trace	0.36	0.97	-0.14	0.93

Table S8 – Performance of cognitive decline models when CAG is added as a feature. Corresponds to Tables 2 and 3 in main text.
